# Supplementary material for: How COVID-19 Pandemic Has Influenced Public Interest in Foods: A Google Trends Analysis of Italian Data
Source: Int J Environ Res Public Health. 2023 Jan 20;20(3):1976. doi: 10.3390/ijerph20031976 (PMC9915381; doi:10.3390/ijerph20031976)
Supplement: Supplementary file 1 [file ijerph-20-01976-s001.zip › ijerph-2115896-supplementary.pdf]

Figure S1. Joinpoint analysis of the public interest in nuts and seeds

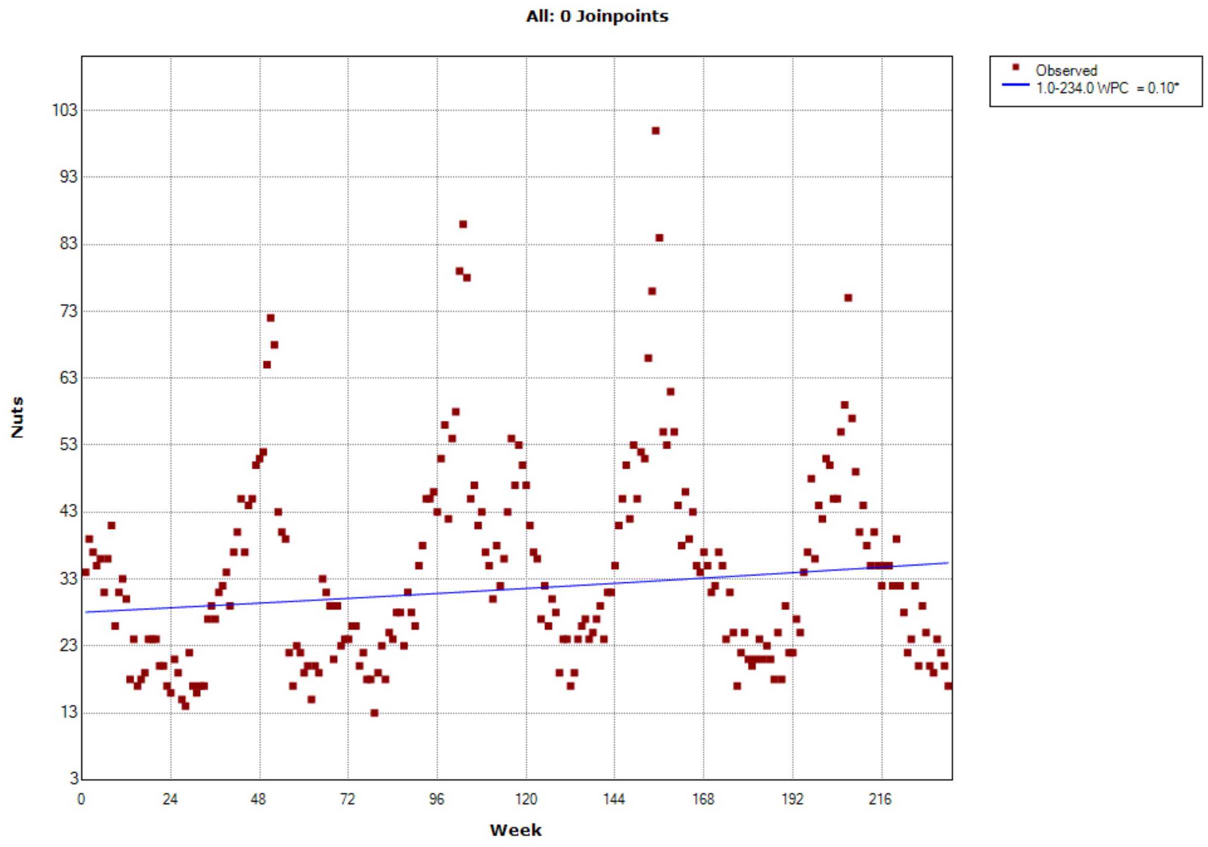

* Indicates that the Weekly Percent Change (WPC) is significantly different from zero at the alpha = 0.05 level.

Figure S2. Joinpoint analysis of the public interest in processed meat

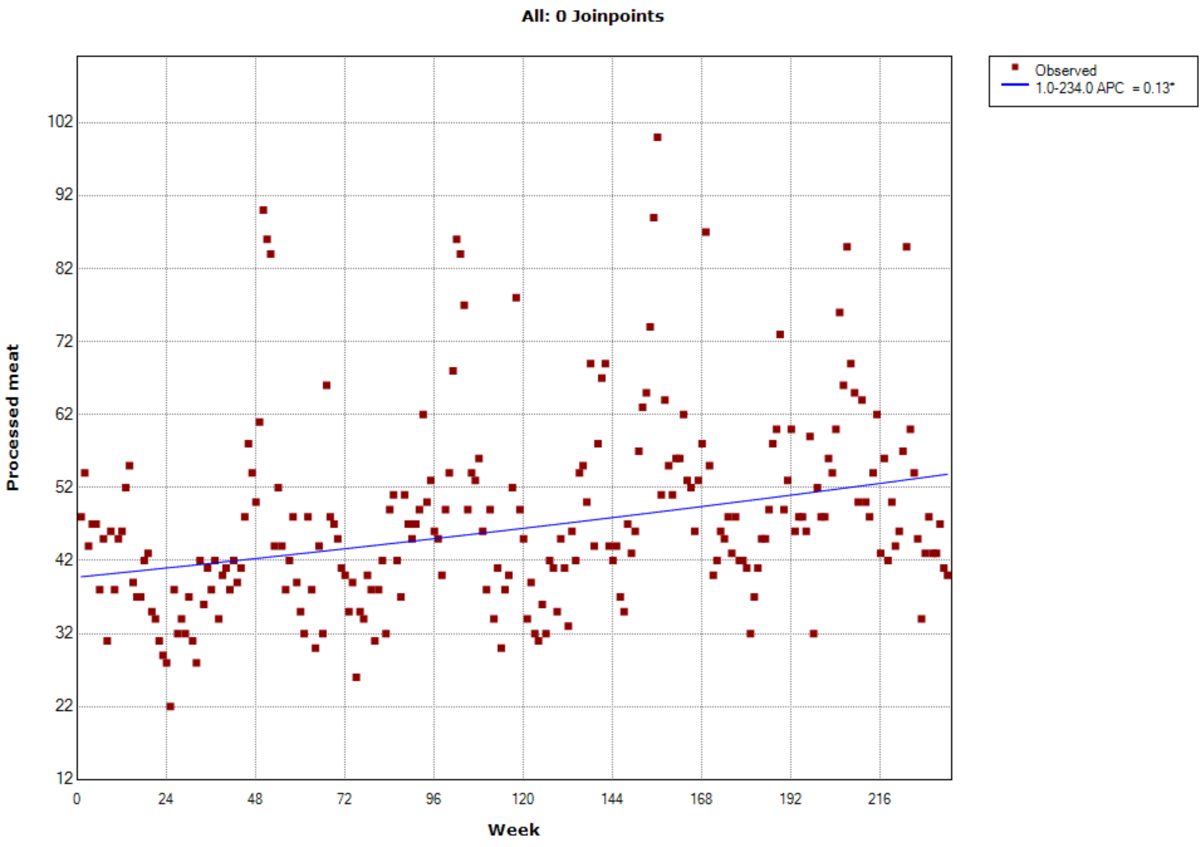

* Indicates that the Annual Percent Change (APC) is significantly different from zero at the alpha = 0.05 level.

Figure S3. Joinpoint analysis of the public interest in sugar sweetened beverages

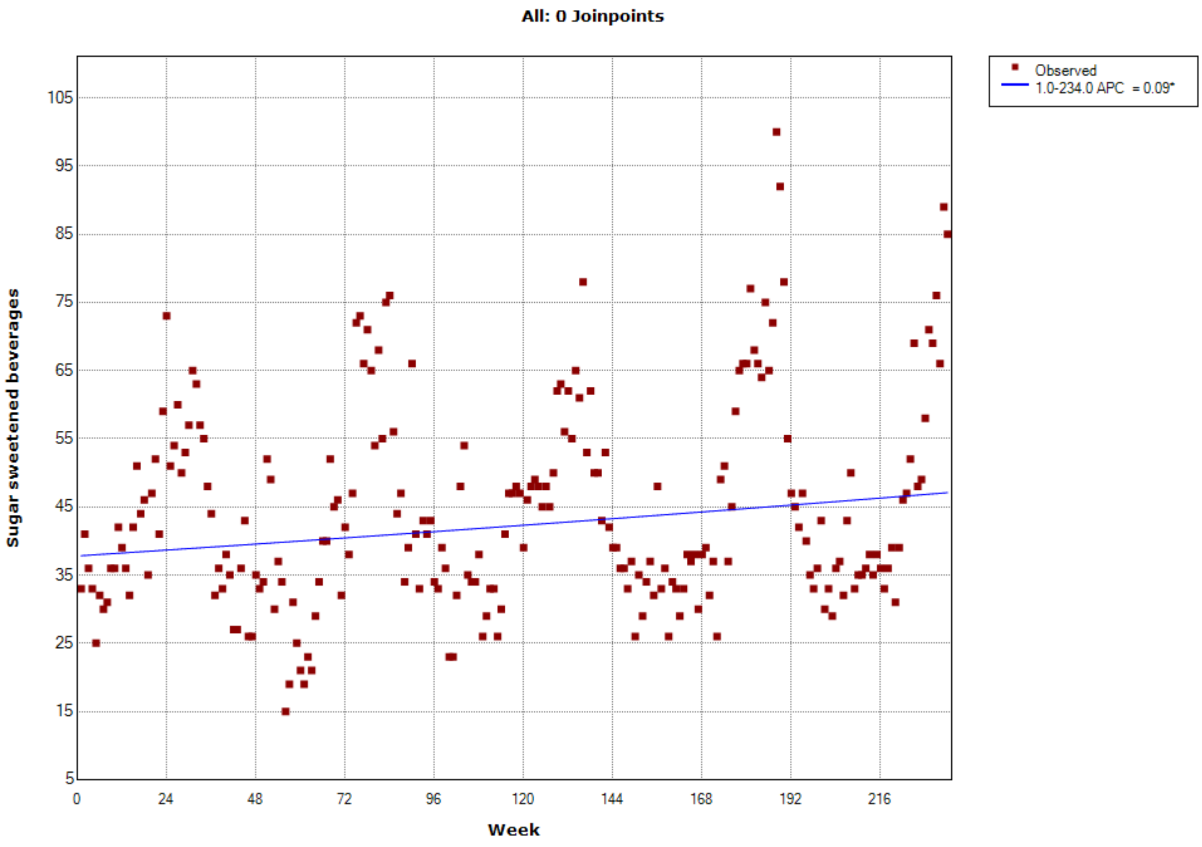

* Indicates that the Annual Percent Change (APC) is significantly different from zero at the alpha = 0.05 level.
